# Src inhibitor reduces permeability without disturbing vascularization and prevents bone destruction in steroid-associated osteonecrotic lesions in rabbits

**DOI:** 10.1038/srep08856

**Published:** 2015-03-09

**Authors:** Yi-Xin He, Jin Liu, Baosheng Guo, Yi-Xiang Wang, Xiaohua Pan, Defang Li, Tao Tang, Yang Chen, Songlin Peng, Zhaoxiang Bian, Zicai Liang, Bao-Ting Zhang, Aiping Lu, Ge Zhang

**Affiliations:** 1 Institute for Advancing Translational Medicine in Bone & Joint Diseases, School of Chinese Medicine, Hong Kong Baptist University, Hong Kong SAR, China; 2 Hong Kong Baptist University Branch of State Key Laboratory of Chemo/Biosensing and Chemometrics of Hunan University, Hong Kong SAR, China; 3 Shum Yiu Foon Shum Bik Chuen Memorial Centre for Cancer and Inflammation Research, Hong Kong Baptist University, Hong Kong SAR, China; 4 Institute of Integrated Bioinformedicine & Translational Science, HKBU Shenzhen Research Institute and Continuing Education, Shenzhen, China; 5 Academician Chen Xinzi Workroom for Advancing Translational Medicine in Bone & Joint Diseases, Kunshan RNAi Institute, Kunshan Industrial Technology Research Institute, Kunshan, Jiangsu, China; 6 Hong Kong Baptist University - Northwestern Polytechnical University Joint Research Centre for Translational Medicine on Musculoskeletal Health in Space, Shenzhen, China; 7 Department of Diagnostic Radiology and Organ Imaging, The Chinese University of Hong Kong, Hong Kong SAR, China; 8 Department of Orthopedics, Second Hospital of Medical College of Ji Nan University, Shenzhen People's Hospital, 518020 Shenzhen, China; 9 Institute of Basic Research in Clinical Medicine, China Academy of Chinese Medical Sciences, Beijing, China; 10 Department of Obstetrics & Gynaecology, The Chinese University of Hong Kong, Hong Kong SAR, China; 11 Department of Orthopaedics and Traumatology, BaoAn Hospital affiliated to Southern Medical University & Shenzhen 8th People Hospital, Shenzhen, PR China; 12 School of Chinese Medicine, The Chinese University of Hong Kong, Hong Kong SAR, China

## Abstract

To examine the therapeutic effect of Src inhibitor on the VEGF mediating vascular hyperpermeability and bone destruction within steroid-associated osteonecrotic lesions in rabbits. Rabbits with high risk for progress to destructive repair in steroid-associated osteonecrosis were selected according to our published protocol. The selected rabbits were systemically administrated with either Anti-VEGF antibody (Anti-VEGF Group) or Src inhibitor (Src-Inhibition Group) or VEGF (VEGF-Supplement Group) or a combination of VEGF and Src inhibitor (Supplement & Inhibition Group) or control vehicle (Control Group) for 4 weeks. At 0, 2 and 4 weeks after administration, *in vivo* dynamic MRI, micro-CT based-angiography, histomorphometry and immunoblotting were employed to evaluate the vascular and skeletal events in different groups. The incidence of the destructive repair in the Anti-VEGF Group, Src-Inhibition Group and Supplement & Inhibition Group was all significantly lower than that in the Control Group. The angiogenesis was promoted in VEGF-Supplement Group, Src-Inhibition Group and Supplement & Inhibition Group, while the hyperpermeability was inhibited in Anti-VEGF Group, Src-Inhibition Group and Supplement & Inhibition Group. The trabecular structure was improved in Src-Inhibition Group and Supplement & Inhibition Group. Src inhibitor could reduce permeability without disturbing vascularization and prevent destructive repair in steroid-associated osteonecrosis.

Pulsed steroids are frequently prescribed for infectious diseases (e.g. Severe Acute Respiratory Syndrome, SARS) for life-saving and rheumatoid diseases (e.g. Systemic Lupus Erythematosus, SLE) for disease-modifying, respectively^1^,^2^. Inevitably, steroid-associated osteonecrosis commonly occurs^3^. Subchondral collapse is an advanced stage of osteonecrosis that is challenging to our orthopedic surgeons as surgical prognosis of total joint replacement for treatment of collapsed joint is poor^4^.

The subchondral collapse is directly attributed to the dominant destructive repair, whereas no subchondral collapse is found in osteonecrotic patients undergoing reparative osteogenesis without destructive repair. The clinical bioimaging data have demonstrated that the histopathological features of the destructive repair in steroid-associated osteonecrosis can be characterized as continuous marrow edema (vascular event) closely coupled with persistent bone resorption (skeletal event)^5^,^6^. Our previous work has already established a steroid-associated ON rabbit model with dominant destructive repair, and we observed high VEGF expression in the rabbits with dominant destructive repair^7^.

VEGF, first described as “vascular permeability factor”, contributes to tissue edema, as it is expressed within hours following ischemic injury in mouse model^8^. Direct evidence was that intravascular injection of VEGF into healthy mice induced endothelial gaps and subsequent vascular permeability^9^. Also, the VEGF family plays a paramount role in promoting angiogenesis or vasculogenesis, which may be induced by local hypoxic conditions to promote survival, migration, and proliferation of endothelial cells (including EPCs)^10^. So, VEGF may not only be associated with positive revascularization of damaged tissue but also may contribute to edema. On the other hand, in a rat femoral head model of vessel deprivation–induced osteonecrosis, high VEGF expression accounted for the striking bone resorption-related remodeling of necrotic debris early during repair, with subsequent substitution by newly formed bone^11^,^12^. It is known that continuously high VEGF exposure, however, serves as a chemoattractant for osteoclasts to induce osteoclastogenesis for bone resorption *in vitro* through a matrix metalloproteinase 9-dependent mechanism, which is similar to signaling pathways involving RANKL^13^,^14^.

Proto-oncogene tyrosine-protein kinase Src (encoded by the c-src gene) is a non-receptor tyrosine kinase localized to the cellular membrane, involved in the regulation of a range of cellular processes, including proliferation, adhesion, motility and survival^15^. For example, Src, as a downstream molecule of VEGF signaling, participates in mediating VEGF-induced vascular permeability in myocardial infarction mouse model^9^. Generally, Src family kinases (SFKs) representing a family of 9 similar proteins include Src, Blk, Fgr, Fyn, Hck, Lck, Lyn, Yes and Yrk^15^. The reviewed evidence just demonstrated selective requirement for Src kinases during VEGF-induced angiogenesis and vascular permeability. Briefly, mice lacking individual Src family kinases (e.g. Src) showed normal VEGF-induced angiogenesis, while mice deficient in Src showed no VEGF-induced vascular permeability. This suggests that VEGF-mediated angiogenesis requires SFK activity in general, whereas vascular permeability mediated by VEGF specifically depended on Src^16^. On the other hand, Src-deficient osteoclasts display decreased migration and fail to form a polarized ruffled membrane during bone resorption^17^. Furthermore, targeted disruption of Src in mice causes a defect in osteoclast-mediated bone resorption, leading to osteopetrosis^18^. Normal osteoclast function can be rescued by bone-specific expression of Src in Src knockout mice^19^. Similar results have been found *in vitro*^20^,^21^. Suppression of Src also interferes with ion transport, which is required to solubilize bone mineral during bone resorption by osteoclasts^22^.

Our previous study showed that both VEGF and phosphorylated Src expression levels were elevated in ON rabbit model with destructive repair^23^. On the other hand, our unpublished clinical data demonstrated that the VEGF level in serum and the phosphorylated Src expression in bone specimen from patients with osteonecrosis (hip joint replacement due to femoral head avascular necrosis) were remarkably higher than those from patients without osteonecrosis (hip joint replacement due to fracture) (Supplement 1).

Based on the previous research, we put forward our hypothesis that Src inhibitor blocking aberrant VEGF-Src signaling could inhibit both vascular event (continuous marrow edema) and skeletal event (persistent bone resorption) of destructive repair but preserve VEGF-induced neovascularization in steroid-associated osteonecrosis. Our previously established steroid-associated ON rabbit model with dominant destructive repair was employed to test the hypothesis using our published bioimaging modalities and evaluation protocols, including perfusion MRI, microCT-based angiography and trabecular micro-architecture, light microscopy for bone histomorphometry, and immunoblotting of bone marrow tissue^7^,^24^,^25^,^26^,^27^.

## Results

### Incidence of the Destructive Repair in Osteonecrotic Lesions

**Figure 1:**
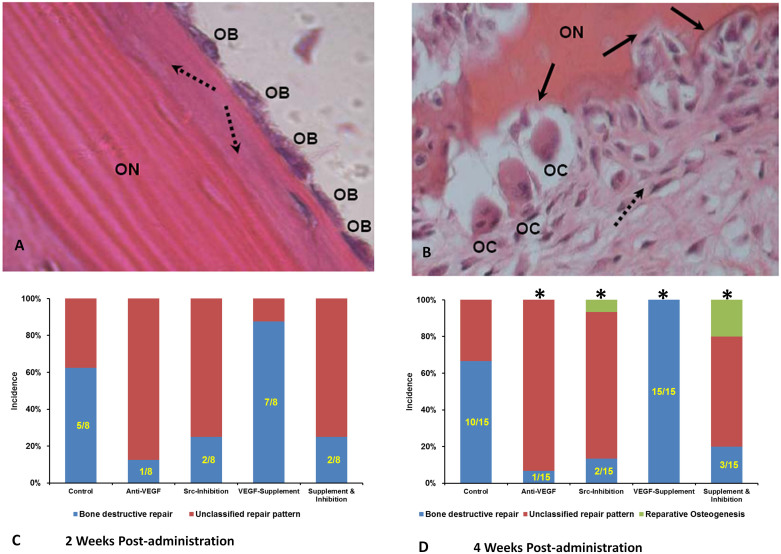
Osteonecrosis Lesion Differentiation and Classification at 2 weeks and 4 weeks Post-administration. (A). Appositional bone formation (dashed arrows) with osteoblasts (OB) around the necrotic bone (ON), defined as ‘reparative osteogenesis’. (B). Eroded bone surface (solid arrows) with osteoclasts (OC) around the necrotic bone (ON) during fibrous tissue creep (dashed arrow), defined as ‘destructive repair’. (C & D). The incidence of the destructive repair in the Anti-VEGF Group, Src-Inhibition Group and Supplement & Inhibition Group was all lower than that in the Control Group, whereas it was higher in the VEGF-Supplement Group when compared to the Control Group at 2 weeks and 4 weeks Post-administration. N = 8 for 2 Weeks Post-administration, N = 15 for 4 Week Post-administration, Fisher's exact probability test, *P < 0.05 vs Control.

At 2 weeks after administration (4 weeks post-induction), the incidence of the destructive repair in the Anti-VEGF Group (1/8), Src-Inhibition Group (2/8) and Supplement & Inhibition Group (2/8) was all lower than that in the Control Group (5/8), whereas it was higher in the VEGF-Supplement Group (7/8) when compared to the Control Group. However, due to the sample size limitation, the difference was not statistically significant. At 4 weeks after administration (6 weeks post-induction), 10 of those 15 rabbits in Control group had dominant destructive repair, whereas 1 of 15, 2 of 15, 15 of 15 and 3 of 15 rabbits had dominant destructive repair in Anti-VEGF Group, Src-Inhibition Group, VEGF-Supplement Group and Supplement & Inhibition Group, respectively. The incidence of the destructive repair in the Anti-VEGF Group, Src-Inhibition Group and Supplement & Inhibition Group was all significantly lower than that in the Control Group, whereas it was significantly higher in the VEGF-Supplement Group (15/15) when compared to the Control Group. There are even 1 and 3 rabbits showed dominant reparative osteogenesis in the Src-Inhibition Group and Supplement & Inhibition Group respectively. The repair pattern in the rest of the rabbits can not be clearly identified. (Figure 1)

### Measurement of Trabecular Structure in Osteonecrotic Lesions

**Figure 2:**
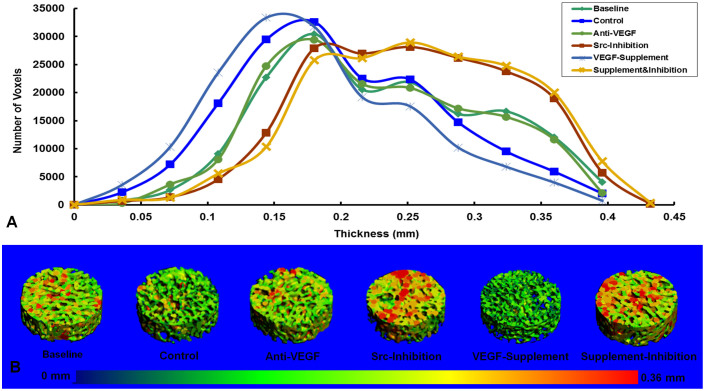
Shift of the trabecular structural profile during osteonecrotic lesion repair in different groups. (A). Size distribution of trabecular bone of osteonecrotic lesion at 4-weeks post-administration in different groups. (B). Representative 3-D structure of trabecular bone of osteonecrotic lesion at 4-weeks post-administration in different groups. N = 7

For trabecular structure of osteonecrotic lesions by micro-CT measurement, there is no difference in quantities of either small-sized (0.036 ~ 0.2 mm) or large-sized (0.2 ~ 0.4 mm) trabecular bone between Anti-VEGF Group and Baseline Group. Compared to that at Baseline, less large-sized and more small-sized trabecular bones were found in Control Group and VEGF-Supplement Group, whereas more large-sized and less small-sized trabecular bone were found in Src-Inhibition Group and Supplement & Inhibition Group. Apparently, the size distribution of the trabeculae shifted toward thinning in the Control Group when compared to the baseline, and it further shifted toward thinning in the VEGF-Supplement Group when compared to the Control Group, whereas it hardly shifted in the Anti-VEGF Group or shifted toward moderately thickening in Src-Inhibition Group and Supplement & Inhibition Group when compared to the baseline. (Figure 2)

### Vascular Function Index

**Figure 3:**
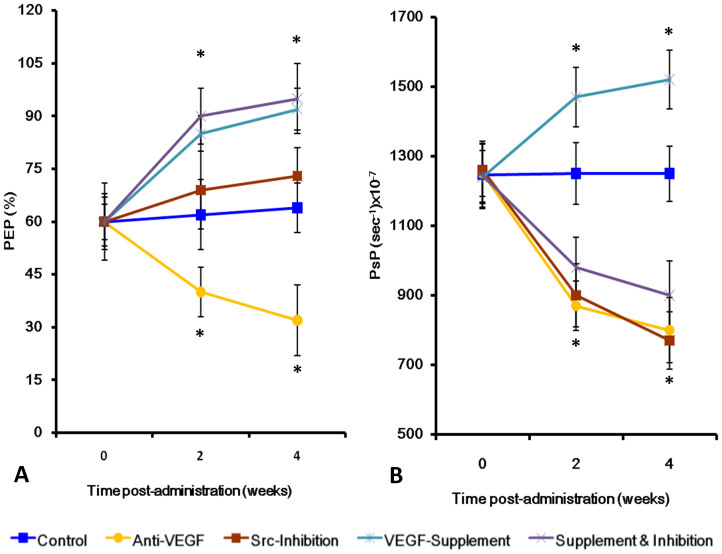
Time course changes in dynamic magnetic resonance imaging (MRI)-derived vascular function indices in different groups. (A). The PEP (vascularization index) in the VEGF-Supplement Group and Supplement & Inhibition Group Increased continuously and significantly from baseline at 2 weeks and 4 weeks post-administration; while it decreased continuously from Baseline in Anti-VEGF Group at 2 weeks and 4 weeks post-administration. (B). The Psρ (Permeability index) in the Src-Inhibition Group, Supplement & Inhibition Group and Ant-VEGF Group decreased continuously and significantly from baseline at 2 weeks and 4 weeks post-administration; while it increased continuously from Baseline in VEGF-Supplement Group at 2 weeks and 4 weeks post-administration. N = 15, Two-way repeat measure ANOVA, *P < 0.05 vs Control at corresponding time point.

Time-course changes in dynamic MRI–derived vasculature function index are shown in Figure 3. For the vascularization index (peak enhancement percentage, PEP), a significantly different pattern of PEP change over time in VEGF-Supplement Group, Supplement & Inhibition Group and Anti-VEGF Group compared to Control Group was evidenced by the Two-way repeat measures ANOVA (P < 0.05 for interaction between ‘Time’ and ‘Group’). The PEP in the VEGF-Supplement Group and Supplement & Inhibition Group increased continuously and significantly from baseline at 2 weeks and 4 weeks post-administration (25% for VEGF-Supplement Group, 30% for Supplement & Inhibition Group at week 2 and 32% for VEGF-Supplement Group, 35% for Supplement & Inhibition Group at week 4, P < 0.05 for all); while it decreased continuously from Baseline in Anti-VEGF Group at 2 weeks and 4 weeks post-administration (−20% at week 2 and −28% at week 4, P < 0.05 for both).It remained almost constant in the Control Group and increased gradually without significance in Src-Inhibition Group.

For the permeability index (‘permeability surface area product per unit volume of tissue’, PSρ), a significantly different pattern of PSρ change over time in VEGF-Supplement Group, Src-Inhibition Group, Supplement & Inhibition Group and Ant-VEGF Group compared to Control Group was evidenced by the General Linear Model (GLM) (P < 0.05 for interaction between ‘Time’ and ‘Group’). The PSρ in the Src-Inhibition Group, Supplement & Inhibition Group and Ant-VEGF Group decreased continuously and significantly from baseline at 2 weeks and 4 weeks post-administration (−29% for Src-Inhibition Group, −21% Supplement & Inhibition Group and −30% for Ant-VEGF Group at week 2 and −39% for Src-Inhibition Group, −28% Supplement & Inhibition Group and −36% for Ant-VEGF Group P < 0.05 for all); while it increased continuously from Baseline in VEGF-Supplement Group at 2 weeks and 4 weeks post-administration (19% at week 2 and 23% at week 4, P < 0.05 for both).It remained almost constant in the Control Group (Figure 3).

### Micro-CT based Angiography of Intraosseous Vascularture

**Figure 4:**
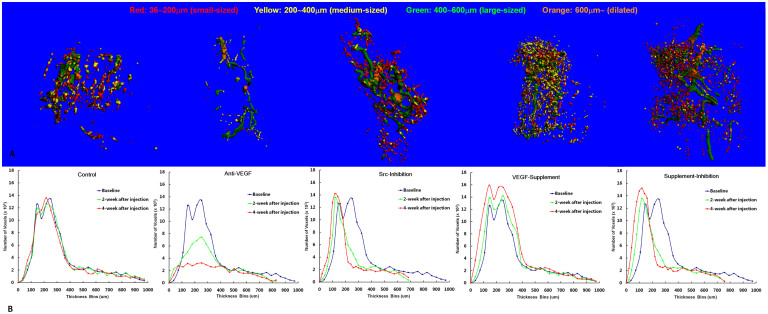
Angiographic analyses of the size and thickness of the vessel structures in the bilateral proximal femora of rabbits. (A). Representative 3-D angiogram at 4 weeks post-administration in different groups. (B) Size distribution of angiographic structure 4 weeks post-administration in different groups. N = 8

Figure 4 presents representative 3-D angiograms and histograms depicting the size of angiographic structures. The Control Group showed large-sized (400 ~ 600 μm) vessel-like structures (VLS) surrounded by both fewer small-sized (36 ~ 200 μm) VLS and many medium-sized (200 ~ 400 μm) disseminated leakage particle–like structures (DLPLS); The Anti-VEGF Group showed only dilated and large-sized VLS, but neither small-sized VLS nor medium-sized DLPLS; The Src-Inhibition Group showed some dilated and large-sized VLS surrounded by more small-sized VLS but no medium-sized DLPLS compared to Control Group; In the VEGF-Supplement Group, although there are more small-sized VLS, but there are also more medium-sized DLPLS compared to the control; In the Supplement & Inhibition Group, there are more small-sized VLS with nearly no medium-sized DLPLS compared to Control Group (Figure 4A).

In the histogram, the Control Group showed neither a continuous increase in small-sized VLS nor a continuous decrease in DLPLS when compared with the baseline. A continuous decrease in medium-sized DLPLS after administration in the Anti-VEGF Group, Src-Inhibition Group and Supplement & Inhibition Group was found with similar changing pattern. A continuous slight increase in small-sized VLS after administration was found in the Src-Inhibition Group, and a continuous significant increase in small-sized VLS after administration was found in both the VEGF-Supplement Group and Supplement & Inhibition Group, whereas a continuous decrease in small-sized VLS was only found in the Anti-VEGF Group (Figure 4B).

### Histomorphometry of Marrow Circulation

**Figure 5:**
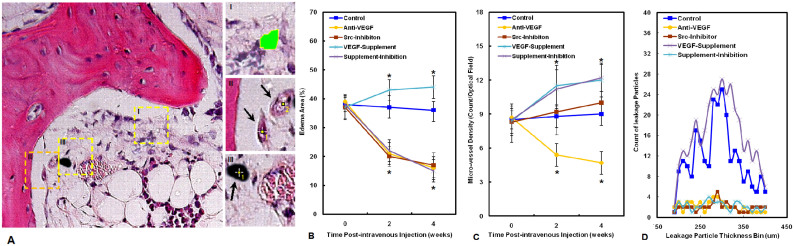
Histomorphometric analyses of the bone marrow circulation. (A). Analysis procedure of edema area (region I in the left image and indicated by green color in the right enlarged image), micro vessel density (region II in the left image and indicated by arrows in the right enlarged image) and size distribution of leakage particles (region III in the left image and indicated by arrow in the right enlarged image) in different groups using Image J software. (B). Time-course change in edema area post-administration in different groups. (C). Time-course change in micro vessel density post-administration in different groups. (D). Comparison of size distribution of leakage particles in different groups. N = 8, One-way ANOVA with Student-Newman-Keuls post hoc test, *P < 0.05 vs Control at corresponding time point.

Figure 5 presents histomorphometry of marrow circulation at 0, 2 4 weeks post administration, including micro-vessel density (MVD), edema area (EA), and leakage particle size distribution. EA in the Anti-VEGF Group, Src-Inhibition Group and Supplement & Inhibition Group decreased continuously and significantly with similar changing pattern from the baseline after administration, whereas it was almost remained in the Control Group and even increased continuously and significantly in the VEGF-Supplement Group (Figure 5B). MVD in the Anti-VEGF Group decreased continuously and significantly from the baseline after administration, whereas it was almost maintained in the Control Group, increased slightly and continuously in the Src-Inhibition Group, and even increased continuously and significantly in the VEGF-Supplement Group and Supplement & Inhibition Group (Figure 5C). In comparison with the Control Group, leakage particles were less found in the Anti-VEGF Group, Src-Inhibition Group and Supplement & Inhibition Group, whereas there were a lot of leakage particles in the VEGF-Supplement Group (Figure 5D).

### Expression of phosphorylated Src and total Src in Bone Marrow

**Figure 6:**
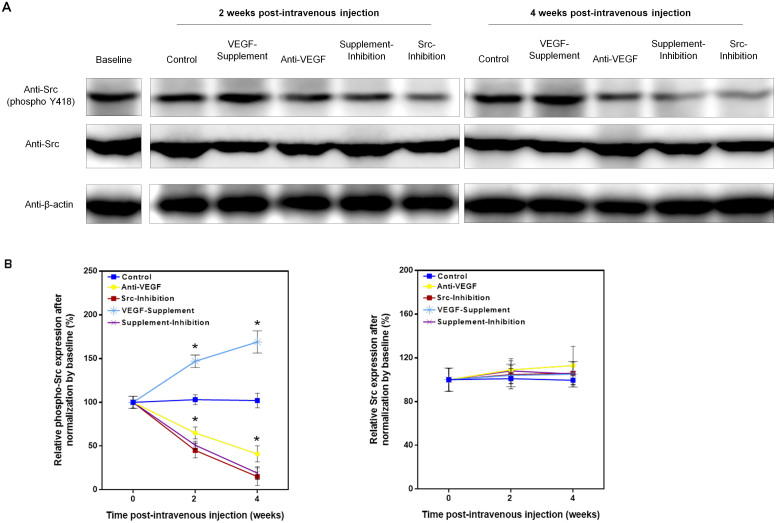
Expression of phosphorylated Src and total Src in bone marrow in different groups. (A). Representative electrophoresis bands for bone marrow phosphorylated Src and total Src expression at baseline and in each treatment group at 2 and 4 weeks post-intravenous injection. (B). Time-course changes in bone marrow phosphorylated Src expression and total Src expression in each group. N = 8, One-way ANOVA with Student-Newman-Keuls post hoc test, *P < 0.05 vs Control at corresponding time point.

Phosphorylated Src protein expression in the Anti-VEGF Group, Src-Inhibition Group and Supplement & Inhibition Group was decreased continuously and significantly from the baseline with similar changing pattern after administration, whereas it did not change in the Control Group or further significantly increased in the VEGF-Supplement Group. On the other hand, the total Src expression level remained stable from baseline to 4 weeks post administration, and did not show difference among groups (Figure 6).

## Discussion

This study specifically investigated the effect of Src inhibitor on the VEGF mediating vascular hyperpermeability and bone destruction within steroid-associated osteonecrotic lesions in rabbits with low-level marrow stem-cell-pool (MSCP) after initial osteonecrotic lesion formation. Results from the dynamic MRI perfusion function index, Micro-CT-based angiography, and histomorphometry of marrow circulation consistently demonstrated that anti-VEGF reduced both neovascularization and permeability, whereas a Src inhibitor did not reduce neovascularization but did reduce permeability. In addition, immunoblotting for phosphorylated Src also demonstrated significantly decreased Src phospho-Y418 levels in the Src-Inhibition, Anti-VEGF and Supplement & Inhibition Groups at each post-administration time point. Taken together, these results suggest that uncontrolled VEGF-Src signaling underlies the observed continuous increase in vascular permeability during inadequate repair of steroid-associated osteonecrosis, which is consistent with a recent consensus that normal vascular turnover requires precise spatiotemporal control of VEGF expression^28^. Accordingly, it encourages use of a selective blockade strategy of Src signaling for both maintaining VEGF-mediated angiogenesis and abolishing VEGF-mediated permeability to facilitate repair.

The histopathological results from lesion classification in this study showed that anti-VEGF inhibited destructive repair after osteonecrotic lesion formation, as evidenced by both significantly reduced incidence of destructive repair and the no significantly shifted size distribution curve of trabecular thickness in Anti-VEGF Group compared to the Baseline. In contrast, VEGF promoted destructive repair as evidenced by 100% incidence of destructive repair and moderately left shifted size distribution curve of trabecular thickness in VEGF-Supplement Group compared to the Control. VEGF is a chemoattractant for osteoclasts to induce osteoclastogenesis *in vitro* and *in vivo* through a matrix metalloprotease 9–dependent mechanism, which is similar to signaling pathways involving receptor activator of NF-kappaB ligand (RANKL)^13^,^29^,^30^,^31^. These data suggest a potential link between uncontrolled VEGF signaling and destructive repair of steroid-associated osteonecrotic lesions for testing a therapeutic strategy by blocking uncontrolled VEGF signaling, which not only challenges the traditionally held opinion that enhanced VEGF signaling might augment bone repair, but also raises an emerging concept that uncontrolled VEGF signaling could induce destructive repair when MSCP is at a continuously low level.

On the other hand, the study showed that a Src inhibitor moderately promoted reparative osteogenesis after osteonecrotic lesion formation, as evidenced by both the moderately increased incidence of reparative osteogenesis and moderately right shifted size distribution curve of trabecular thickness in the Src-Inhibition and Supplement & Inhibition Groups as compared with the Control. This could be explained by the significantly reduced vascular permeability caused by the Src inhibitor to avoid diverting blood away from the lesion center towards its periphery and accordingly facilitating delivery of oxygen and nutrients to local lesions for tissue reconstructional repair^32^. These results also suggest a potential causal relationship between continuous hyperpermeability and inactive reparative osteogenesis for testing a therapeutic strategy via blockade of uncontrolled Src signaling.

This time, we used a Src inhibitor PP1 to modulate the phosphorylated Src expression level, and further examined the vascular and skeletal impact of the intervention. PP1 (4-amino-5-(4-methylphenyl)-7-(t-butyl)pyrazolo(3,4-d)pyrimidine) is a cell-permeable pyrazolopyrimidine compound that inhibits Src activity with IC50 of 170 nM^33^. Dr. Weis et al. used it to block Src Y418 phosphorylation by intravenous injection in mice^9^. Dr.Zan et al. injected PP1 in to a focal cerebral ischemia rat model, and demonstrated that PP1 effectively decreased Src Y418 phosphorylation level and reduced the vascular permeability in the rat brain^34^. In the present study, PP1 was intravenously administrated at a dose of 0.3 mg/kg according to dose conversion principle among different animals^35^,^36^, which corresponded to the effective dose for anti-permeability in previous two studies. The efficacy of systemic administration of PP1 was also verified in this study, as evidenced by significantly decreased phosphorylated Src expression in Src-Inhibition Group and Supplement & Inhibition Group.

Putting together, we demonstrated Supplement & Inhibition Group showed sound repair outcome, as demonstrated by lower destructive repair incidence, thicker trabecular structure profile, better neovascularization, and lower permeability compared to Control Group. It suggested that supplement of VEGF while inhibiting Src could be a new therapeutic strategy for steroid-associated osteonecrotic patients with high risk of subchondral collapse.

## Methods

### Experimental Design

Male 28-week-old New-Zealand white rabbits with body weight of 4 ~ 5 kg were housed at the Animal house in Institute for Advancing Translational Medicine in Bone & Joint Diseases in Hong Kong Baptist University and received a standard laboratory diet and water *ad* *libitum*. All experimental protocols were approved by the Animal Experiment Ethics Committee of Institute for Advancing Translational Medicine in Bone & Joint Diseases in Hong Kong Baptist University (Ref No.TMBJ/12/NR). The methods were carried out in accordance with the approved guidelines and the surgical operation was offcially approved by Hong Kong government (Ref No. (12–30) in DH/HA&P/8/2/6 Pt.2). Based on our established protocol for inducing steroid-associated osteonecrosis development^7^,^24^,^25^,^26^, all the rabbits were intravenously injected once with 10 μg/kg body weight of lipopolysaccharide (*Escherichia coli* 0111:B4, Sigma-Aldrich, USA) on day 0. After 24 hours, three injections of 20 mg/kg body weight of methylprednisolone (Pharmacia & Upjohn, USA) were given intramuscularly at a 24-hour interval. At 0 (pre-induction/baseline) and 1 week after induction, bone marrow aspiration from iliac crest was conducted to determine size of marrow stem cell pool (SI-MSCP) in hematopoietic and mesenchymal compartment according to our published protocol^7^. At 2 weeks after induction, 121 rabbits with a decrease of at least 70% in SI-MSCP of both mesenchymal and hematopoietic compartment were identified as high riskers for progress to destructive repair within osteonecrotic lesions according to the published findings^7^. The selected rabbits were systemically administrated by intravenous injection with either Anti-VEGF antibody (recombinant humanized monoclonal anti-VEGF at 33 mg/kg/two weeks, Anti-VEGF Group, n = 23) or VEGF (recombinant human VEGF at 0.05 mg/kg/two weeks, VEGF-Supplement Group, n = 23) or Src inhibitor (PP1, selective inhibitor of Src activity with IC_50_ of 170 nM, 0.3 mg/kg/two weeks), Src-Inhibition Group, n = 23) or a combination of VEGF and Src inhibitor (recombinant human VEGF at 0.05 mg/kg/two weeks and Src inhibitor PP1 at 0.3 mg/kg/two weeks, Supplement & Inhibition Group, n = 23) and control vehicle (Saline, Control Group, n = 23) for 4 weeks. Six rabbits were sacrificed as baseline before administration. The sample size in each group was calculated according to our published paper^7^. At 0, 2 and 4 weeks after administration, *in vivo* dynamic MRI was performed on proximal femora for vascularization index and permeability index, respectively. After finishing dynamic MRI scan, euthanasia was also executed at 2 (n = 8) and 4 (n = 15) weeks after administration in each group. Bilateral proximal femora were dissected after sacrifice for the following evaluation on intraosseous vasculature, including three-dimensional angiography by micro-CT and two-dimensional histomorphometry of marrow circulation by optical microscopy. Repair pattern of osteonecrotic lesions was both qualitatively classified by histopathology and quantificationally differentiated by micro-CT. Local phosphorylated Src protein expression was quantified by immunoblotting.

### Pre-euthanasia Evaluation on Vascular Function

For Dynamic MRI–derived vascular function index, rabbits were anesthetized with 2.5% sodium pentobarbital (0.4 ml/kg) and then placed in the prone position with lower limbs flexed for MRI scanning using a 1.5-T superconducting system (ACS-NT Intera; Philips Medical Systems, Best, The Netherlands) with a maximum gradient strength of 30 mT/m. A bolus of dimeglumine gadopentetate (Magnevist; Schering, Berlin, Germany) (0.3 mmol/kg/body weight) was rapidly injected by an automatic pump linked to a previously placed 21-gauge catheter into an auditory vein. Dynamic MRI scans were performed in the prescribed plane with the following parameters: short T1-weighted gradient echo sequence, TR/TE = 4/1.4 msec, flip angle = 15, slice thickness = 5 mm, in-plane resolution = 0.86 × 0.86 mm, average = 1. The temporal resolution was approximately 0.6 s per image acquisition. A series of dynamic images were obtained in 600 s to cover the wash-out phase^3^,^24^,^37^,^38^. The vascularization index ‘Peak Enhancement Percentage’ (PEP) and permeability index ‘Permeability Surface Area Product per Unit Volume of Tissue’ (PSρ) were accordingly calculated using our established protocol^24^,^25^.

### Post-euthanasia Evaluation

Under general and deep anesthesia with 2.5% sodium pentobarbital by intravenous instilment (0.4 ml/kg), the rabbit abdominal cavity was opened for perfusion with a confected radiopaque silicone rubber with a combination of neutral buffered formalin (10%) and heparinized normal saline (50 U/ml) using our established protocol^24^,^25^,^39^,^40^. Then, trabecular structure of osteonecrotic lesion in bilateral proximal femoral samples was quantificationally differentiated by micro-CT. After that, the completely decalcified proximal femoral samples by ethylenediaminetetraacetic acid were subjected to Micro-CT-based angiography. Thereafter, the decalcified proximal femur was embedded in paraffin and sectioned at 6 μm thickness along the coronal plane to classify the osteonecrotic lesion repair process with histomorphometry of marrow circulation by OM and local phosphorylated Src by expression by immunoblotting, respectively.

Quantificational Differentiation of Trabecular Structure in Osteonecrotic Lesion: Proximal parts of bilateral un-decalcified femoral samples were taken for measurement of trabecular structure in osteonecrotic lesion using our established protocol^24^,^25^. A histogram was generated to display the size (thickness) distribution of trabecular bone. A color-coded scale was mapped to the surface of the 3-D image to produce a visual representation of the size distribution of trabecular bone^24^,^39^,^41^.

Classification of Osteonecrotic Lesion Repair: Classification of osteonecrotic lesion repair was blindly made by two pathologists using OM (Aixoplan with Spot RT digital camera, Zeiss, Germany). Osteonecrotic lesion formation was identified based on diffuse presence of empty lacunae or pyknotic nuclei of osteocytes in bone trabeculae, accompanied by surrounding bone marrow necrosis^42^. Appositional bone formation with osteoblast-like cells around the osteonecrotic lesion was classified as ‘Reparative Osteogenesis’, whereas granulation tissue creep linked to necrotic bone resorption was classified as ‘Destructive Repair’^43^. Rabbits with no dominant ‘Reparative Osteogenesis’ or ‘Destructive Repair’ were termed “unclassified”.

Micro-CT-based Micro-angiography for Vascular Architecture: Proximal parts of bilateral decalcified femoral samples were taken for intraosseous 3-D Micro-CT-based micro-angiography using our established protocol^24^,^25^. A histogram was generated to display the size (thickness) distribution of angiographic structure. A color-coded scale was mapped to the surface of the 3-D image to produce a visual representation of the size distribution of angiographic structures^24^,^39^,^41^.

Histomorphometry of Marrow Circulation: For micro-vessel density (MVD), fifteen successive hematoxylin and eosin–stained 6-μm-thick sections from every decalcified sample were scanned initially at low magnification and then at high magnification to identify vascular ‘hot-spots’^44^,^45^ using the Optical Microscope imaging system (Zeiss). Selection of the hot-spot has been adopted as a standard procedure for angiogenesis studies in both solid neoplasms and for hematological oncology^46^. It is thought that such areas of increased concentration of micro-vessels may represent the emergence of a neoplastic clone with a higher angiogenic potential^47^. For each countable micro-vessel, an outline was traced to calculate the total count of micro-vessels per total examined optical fields in those successive sections from bilateral decalcified femoral samples in one rabbit (Micro-vessel Density) using image analysis software (ImageJ 1.32j, NIH, USA). For edema area (EA), the above histological sections were scanned to identify the interstitial marrow edema zone by OM. For each section, four randomly selected fields (up, down, left, right) were examined. The marrow edema zone was automatically traced by thresholding using ImageJ 1.32j^24^. The total area of the edema zone per total examined optical field area in those successive sections from bilateral decalcified femoral samples (Edema Area) in one rabbit were calculated accordingly. For size distribution of leakage particles, the above sections were examined with OM to identify leakage particles in the region corresponding to the scanned volume of interest during the above-mentioned Micro-CT-based micro-angiography. An easily distinguished black radiopaque particle (perfused angiographic substance) outside of a blood vessel was defined as a leakage particle. For each leakage particle, the outline was manually traced to quantify its area and perimeter using ImageJ 1.32j. Based on the stereology principle in bone histomorphometry^48^, leakage particle size was calculated as follows: Thickness = 2000/1.199 × (Area/Perimeter). A histogram to display the size distribution of all the leakage particles in those successive sections from bilateral decalcified femoral samples in one rabbit was generated using Excel 2007 (Microsoft, USA).

Local Marrow phosphorylated Src protein and total Src protein expression by Immunoblotting: Marrow tissues were harvested and lysed in homogenization RIPA Lysis Buffer (R2031-75, United States Biological, USA). Homogenates were pre-centrifuged at 2,500 g for 10 min at 4°C, and the collected supernatant was centrifuged at 105,000 g for 0.5 h at 4°C. Protein concentration of the tissue lysate was determined by the BCA protein assay kit (#23225, Pierce, USA). Protein (20 μg) was heated at 95°C for 4 min in gel-loading buffer (Sigma), subjected to 10% SDS-PAGE, and then transferred to a PVDF membrane (Bio-Rad, USA) using a TRANS-BLOT SD Semi-dry Transfer Cell (Bio-Rad). After blocking with 5% defatted milk for 0.5 h, the membrane was incubated with primary antibody, i.e., anti-Src phospho-Y418 at 1:500 (Biosource InternationalUSA)^16^, anti-Src at 1:500 (Biosource International, USA) and β-actin (as an internal control) at 1:2000 overnight, followed by incubation with a secondary antibody (anti-goat IgG at 1:1000; Santa Cruz Biotechnology) conjugated to horseradish peroxidase for 1 h. Proteins (Src-phospho-Y418) were visualized by chemiluminescence with the ECL plus Immunoblotting Detection System (Pierce), and then Src-phospho-Y418 and total Src expressions were normalized to the band intensity of β-actin using a molecular imager system (Bio-Rad). Data presented are representative of at least three separate experiments.

### Data analysis

For statistical analysis, data are expressed as the mean ± SD. Data from repeated measurements (MRI-derived vascular function index) was analyzed by Two-way repeated measures ANOVA. Data from non-repeated measurements, including micro vessel density, edema area, and protein expression level, were analyzed by one-way analysis of variance with post hoc multiple comparison tests (Student-Newman-Keuls test when equal variance was assumed, or Games-Howell test when equal variance was not assumed). Fisher's exact probability test was performed to determine the difference in incidence data (reparative osteogenesis and destructive repair) among groups. All statistical analyses were performed using SPSS 10.0 (SPSS, Chicago, IL, USA). The statistical significance level was P < 0.05.

## Author Contributions

G.Z., A.P.L. and B.T.Z. designed the study; Y.X.H., J.L., B.S.G., Y.X.W., X.H.P., D.F.L. and T.T. conducted the experiment; Y.X.H., J.L., B.S.G. and S.L.P. analyzed the data; Y.X.H., J.L., B.S.G. wrote the manuscript; G.Z., A.P.L., B.T.Z., Z.C.L., Z.X.B., S.L.P. and Y.C. revised the manuscript; G.Z., A.P.L. and B.T.Z. approved final version of the manuscript and took responsibility for the integrity of the data.

## Supplementary Material

Supplementary Information Supplementary Dataset 1
