# Supplementary material for: Src inhibitor reduces permeability without disturbing vascularization and prevents bone destruction in steroid-associated osteonecrotic lesions in rabbits
Source: Sci Rep. 2015 Mar 9;5:8856. doi: 10.1038/srep08856 (PMC4352921; doi:10.1038/srep08856)
Supplement: Supplementary Information — Supplementary Dataset 1 [file srep08856-s1.doc]

**Src inhibitor reduces permeability without disturbing vascularization and prevents bone destruction in steroid-associated osteonecrotic lesions in rabbits**

Yi-Xin HE^1,2,3#^, Jin Liu^1,2#^, Baosheng Guo^1,2,3#^, Yi-Xiang Wang^4^, Xiaohua Pan^1,5^, Defang Li ^1, 2, 6^, Tao Tang^1,7^, Yang Chen^8^, Songlin Peng^5^, Zhaoxiang Bian^1, 2^, Zicai Liang^2^, Bao-Ting Zhang^9^*, Aiping Lu ^1, 2, 6^*, Ge Zhang ^1, 2, 3^*

1. Institute for Advancing Translational Medicine in Bone & Joint Diseases, School of Chinese Medicine, Hong Kong Baptist University, Hong Kong SAR, China; Hong Kong Baptist University Branch of State Key Laboratory of Chemo/Biosensing and Chemometrics of Hunan University, Hong Kong, China; Shum Yiu Foon Shum Bik Chuen Memorial Centre for Cancer and Inflammation Research; Institute of Integrated Bioinformedicine & Translational Science, HKBU Shenzhen Research Institute and Continuing Education, Shenzhen, China
2. Academician Chen Xinzi Workroom for Advancing Translational Medicine in Bone & Joint Diseases, Kunshan RNAi Institute, Kunshan Industrial Technology Research Institute, Kunshan, Jiangsu, China
3. Hong Kong Baptist University - Northwestern Polytechnical University Joint Research Centre for Translational Medicine on Musculoskeletal Health in Space, Shenzhen, China
4. Department of Diagnostic Radiology and Organ Imaging, The Chinese University of Hong Kong, Hong Kong SAR, China
5. Department of Orthopedics, Second Hospital of Medical College of Ji Nan University, Shenzhen People's Hospital, 518020 Shenzhen, China
6. Institute of Basic Research in Clinical Medicine, China Academy of Chinese Medical Sciences, Beijing, China,
7. Department of Obstetrics & Gynaecology, The Chinese University of Hong Kong, Hong Kong SAR, China,
8. Department of Orthopaedics & Traumatology, Shenzhen Second People’s Hospital, Shenzhen, China,
9. School of Chinese Medicine, The Chinese University of Hong Kong, Hong Kong SAR, China

*Correspondence: [zhangge@hkbu.edu.hk](mailto:zhangge@hkbu.edu.hk), [aipinglu@hkbu.edu.hk](mailto:aipinglu@hkbu.edu.hk), [zhangbaoting@cuhk.edu.hk](mailto:zhangbaoting@cuhk.edu.hk)

Tel: (852) 3411 2958; Fax: (852) 3411 2902

Address: Jockey Club School of Chinese Medicine Building, 7 Baptist University Road, Hong Kong Baptist University, Kowloon Tong, Kowloon, Hong Kong SAR, China

# Co-first authors

**A**

Anti-Src (phospho Y418)


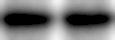


Anti-β-actin

43 kDa

60 kDa


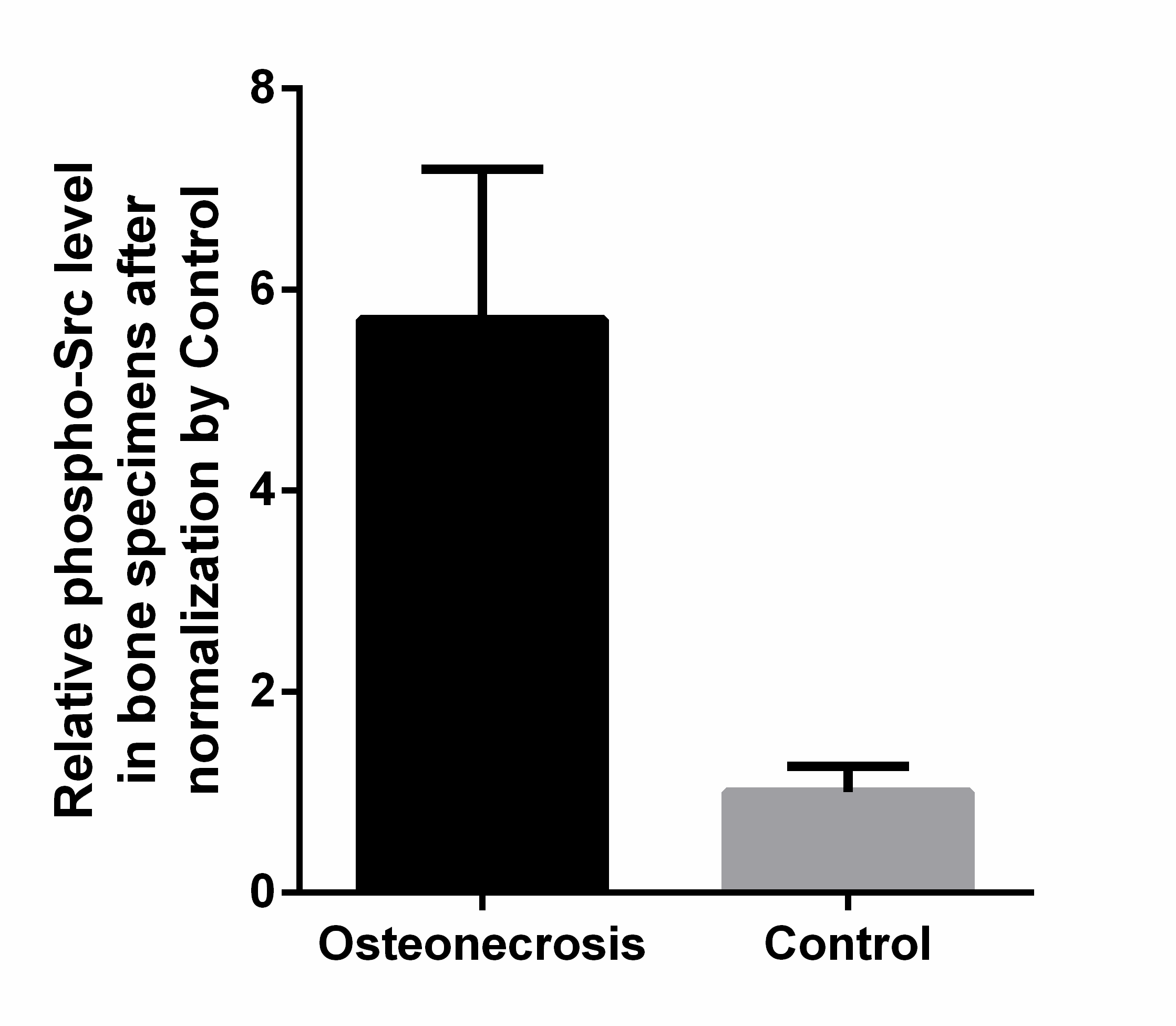

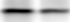


*****


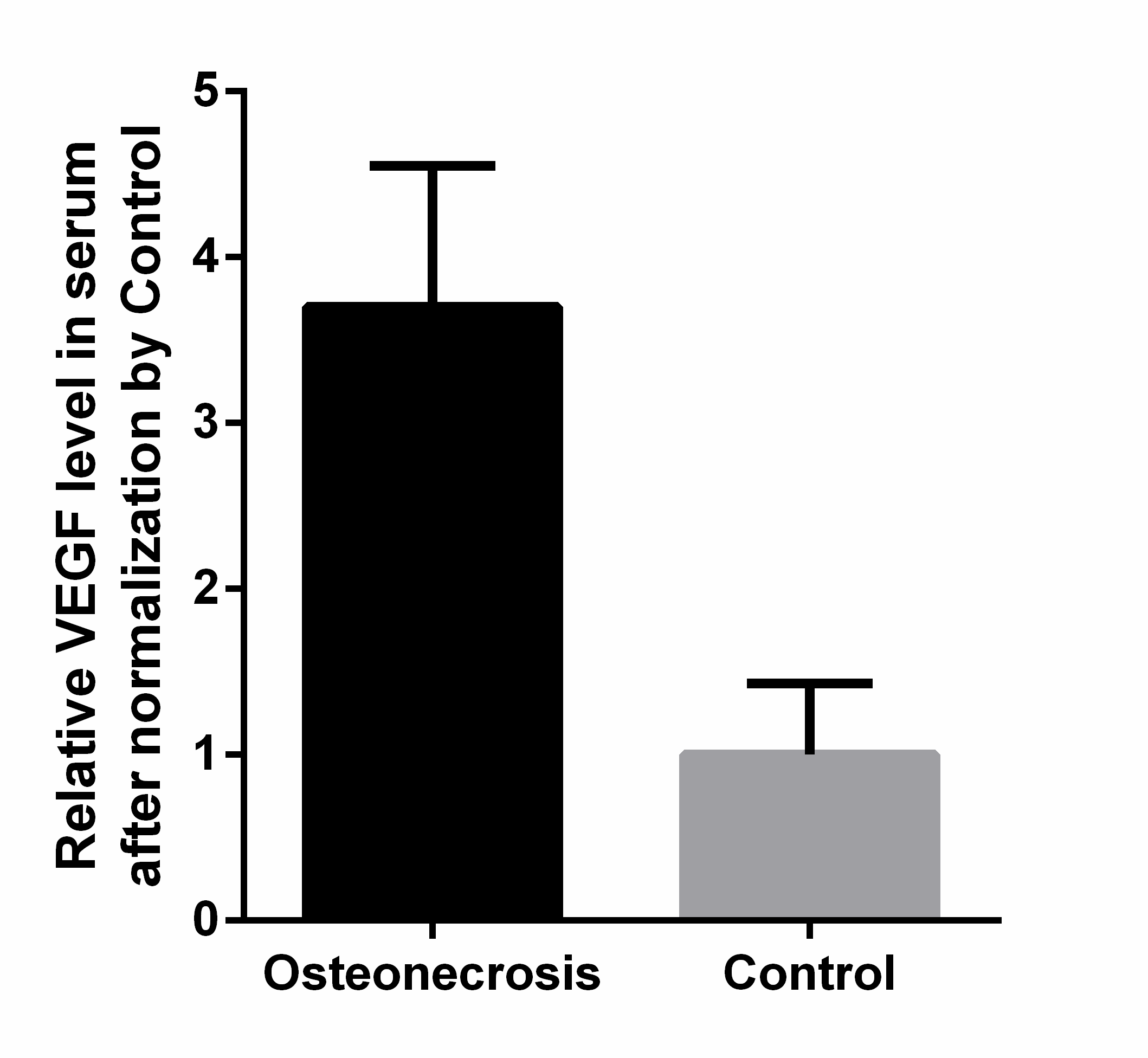


*****

**B**

**Supplement 1. The level of phosphorylated Src in bone specimen and VEGF in serum from patients with or without osteonecrosis receiving joint replacement surgery.** A. The phosphorylated Src level in bone specimens from patients with or without osteonecrosis by western blot analysis. B. The VEGF level in serum from patients with or without osteonecrosis by ELISA.
